# A RAND/UCLA-Modified VAS Study on Telemedicine, Telehealth, and Virtual Care in Daily Clinical Practice of Vascular Medicine

**DOI:** 10.3390/jcm13061750

**Published:** 2024-03-18

**Authors:** Sergio Pillon, Georgia Gomatou, Evangelos Dimakakos, Agata Stanek, Zsolt Pecsvarady, Matija Kozak, Jean-Claude Wautrecht, Katalin Farkas, Gerit-Holger Schernthaner, Mariella Catalano, Aleš Blinc, Grigorios Gerotziafas, Pavel Poredoš, Sergio De Marchi, Michael E. Gschwandtner, Endre Kolossváry, Muriel Sprynger, Bahar Fazeli, Aaron Liew, Peter Marschang, Andrzej Szuba, Dusan Suput, Michael Edmonds, Chris Manu, Christian Alexander Schaefer, George Marakomichelakis, Majda Vrkić Kirhmajer, Jonas Spaak, Elias Kotteas, Gianfranco Lessiani, Mary Paola Colgan, Marc Righini, Michael Lichtenberg, Oliver Schlager, Caitriona Canning, Antonella Marcoccia, Anastasios Kollias, Alberta Spreafico

**Affiliations:** 1 ASL Frosinone, 03100 Frosinone, Italy; sergio.pillon@aslfrosinone.it; 2 Third Department of Medicine, Sotiria General Hospital, National and Kapodistrian University of Athens, 11527 Athens, Greece; edimakakos@yahoo.gr (E.D.); ilkotteas@med.uoa.gr (E.K.); taskollias@gmail.com (A.K.); 3 Department and Clinic of Internal Medicine, Angiology and Physical Medicine, Faculty of Medical Sciences in Zabrze, Medical University of Silesia, 41-808 Zabrze, Poland; astanek@tlen.pl; 4 Second Department of Internal Medicine (Vascular Center), Flor Ferenc Teaching Hospital, 2143 Budapest, Hungary; pecsvarady@gmail.com; 5 Department of Vascular Diseases, Division of Internal Medicine, University Medical Centre, 1000 Ljubljana, Slovenia; 6 Department of Vascular Diseases, Hôpital Erasme, Université Libre de Bruxelles, 1070 Brussels, Belgium; jean.claude.wautrecht@erasme.ulb.ac.be; 7 Department of Angiology, Szent Imre University Teaching Hospital, Tétényi út 12-16, 1115 Budapest, Hungary; farkask@hotmail.com; 8 Department of Medicine II, Division of Angiology, Medical University of Vienna, 1090 Vienna, Austria; 9 Department of Biomedical Science, Inter-University Research Center on Vascular Disease, L. Sacco Hospital, University of Milan, 20157 Milan, Italy; 10 Department of Vascular Diseases, University Medical Centre Ljubljana, 1000 Ljubljana, Slovenia; 11 Department of Internal Medicine, Faculty of Medicine, University of Ljubljana, 1000 Ljubljana, Slovenia; 12 Sorbonne University, Institut National de la Santé et de la Recherche Médicale (INSERM), Unit 938, Research Group Cancer, Biology and Therapeutics, Centre de Recherche Saint-Antoine (CRSA), Institut Universitaire de Cancérologie, 75012 Paris, France; 13 Department for Vascular Disease, University Clinical Center, 1000 Ljubljana, Slovenia; pavel.poredos@kclj.si; 14 Division of Angiology, Department of Medicine, University of Verona, 37134 Verona, Italy; sergio.demarchi@univr.it; 15 Department of Medical Angiology, Medical University Vienna, 1090 Vienna, Austria; michael.gschwandtner@meduniwien.ac.at; 16 Department of Angiology, St. Imre University Teaching Hospital, 1115 Budapest, Hungary; 17 Cardiology Department, University Hospital Liège, 4000 Liège, Belgium; msprynger@gmail.com; 18 Immunology Research Center, Inflammation and Inflammatory Diseases Division, School of Medicine, Mashhad University of Medical Sciences, Mashhad 9177948564, Iran; 19 Portiuncula University Hospital, Soalta University Health Care Group, National University of Ireland Gaway (NUIG), H91 TK33 Galway, Ireland; aaron.liew@nuigalway.ie; 20 Department of Internal Medicine, Central Hospital of Bolzano (SADES-ASDAA), Teaching Hospital of Paracelsus Medical University (PMU), I-39100 Bolzano, Italy; peter.marschang@sabes.it; 21 Department of Angiology and Internal Medicine, Wroclaw Medical University, 50-367 Wroclaw, Poland; 22 Institute of Pathophysiology, Faculty of Medicine, University of Ljubljana, 1000 Ljubljana, Slovenia; dusan.suput@mf.uni-lj.si; 23 Diabetic Foot Clinic, King’s College Hospital, Denmark Hill, London SE5 9RS, UK; 24 Department of Internal Medicine II—Pneumology/Cardiology/Angiology, University Hospital Bonn, 53127 Bonn, Germany; 25 4th Department of Internal Medicine, Evangelismos Hospital, 16676 Athens, Greece; gmarakom@gmail.com; 26 Department of Cardiovascular Diseases, University Hospital Centre Zagreb, University of Zagreb School of Medicine, 10000 Zagreb, Croatia; 27 Department of Clinical Sciences, Danderyd University Hospital, Karolinska Institutet, SE-182 88 Stockholm, Sweden; jonas.spaak@ki.se; 28 Angiology Unit, Villa Serena Hospital, AUSL 3, 65121 Pescara, Italy; 29 Department of Vascular Surgery, St. James Hospital, D08 NHY1 Dublin, Ireland; 30 Division of Angiology and Hemostasis, Faculty of Medicine, Geneva University Hospitals, CH-1211 Geneva, Switzerland; Marc.Righini@hcuge.ch; 31 Vascular Center, Klinikum Hochsauerland, 59759 Arnsberg, Germany; klichte@gmx.net; 32 Department of Internal Medicine II, Division of Angiology, Medical University of Vienna, 1090 Vienna, Austria; 33 Department of Vascular and Endovascular Surgery, St. James’s Hospital D08 NHY1 Dublin, Ireland; 34 UOSD Medicina Vascolare-Autoimmunità-CRIIS Centro di Riferimento Sclerosi Sistemica Osp. S. Pertini-ASL Roma, 00157 Rome, Italy; antonella.marcoccia@aslroma2.it; 35 Digital Health & Innovation, Healthware Group, 20125 Milan, Italy; alberta.spreafico@gmail.com

**Keywords:** telemedicine, telehealth, vascular medicine

## Abstract

**Background:** Telemedicine is increasingly used in several fields of healthcare, including vascular medicine. This study aimed to investigate the views of experts and propose clinical practice recommendations on the possible applications of telemedicine in vascular medicine. **Methods:** A clinical guidance group proposed a set of 67 clinical practice recommendations based on the synthesis of current evidence and expert opinion. The Telemedicine Vascular Medicine Working Group included 32 experts from Europe evaluating the appropriateness of each clinical practice recommendation based on published RAND/UCLA methodology in two rounds. **Results:** In the first round, 60.9% of clinical practice recommendations were rated as appropriate, 35.9% as uncertain, and 3.1% as inappropriate. The strongest agreement (a median value of 10) was reached on statements regarding the usefulness of telemedicine during the 2019 coronavirus disease (COVID-19) pandemic, its usefulness for geographical areas that are difficult to access, and the superiority of video calls compared to phone calls only. The lowest degree of agreement (a median value of 2) was reported on statements regarding the utility of telemedicine being limited to the COVID-19 pandemic and regarding the applicability of teleconsultation in the diagnosis and management of abdominal aortic aneurysm. In the second round, 11 statements were re-evaluated to reduce variability. **Conclusions:** This study highlights the levels of agreement and the points that raise concern on the use of telemedicine in vascular medicine. It emphasizes the need for further clarification on various issues, including infrastructure, logistics, and legislation.

## 1. Introduction

As defined by the American Telemedicine Association (ATA), “Telehealth effectively connects individuals and their healthcare providers when in-person care is not necessary or not possible” [1]. Through telehealth services, patients may receive healthcare, consult with a healthcare provider, access information about a specific condition or treatment, coordinate prescription needs, and obtain a diagnosis [1]. Initially introduced in the 1990s, telehealth has been increasingly used during the past ten years, particularly after the outbreak of the 2019 coronavirus disease (COVID-19) pandemic [2]. The term telemedicine is often used as an alternative to telehealth, encompassing a spectrum of remote clinical services in terms of diagnosis, monitoring, and prescribing therapies employing information and communications technology (ICT) [2]. In everyday clinical practice, the most common utilization of this concept involves teleconsultation, characterized as a synchronous or asynchronous consultation utilizing ICT to overcome geographical and functional barriers, ultimately minimizing disparities in access to healthcare [2,3]. 

Importantly, the ongoing evolution of demographic dynamics, with a growing population of the elderly and patients with chronic diseases, has been redefining the population’s health needs and highlights the necessity to redesign the structural and organizational network of services [4]. For example, it is crucial to strengthen the territorial scope of assistance. Technological innovation is a significant contributor to the reorganization of healthcare by supporting the shift of the focus from the hospital to the territory through innovative, citizen-centered care models and facilitating access to services in inaccessible areas [5]. Telemedicine methods are crucial in promoting equal access to healthcare in remote regions. The ICT tools employed in telemedicine services enable access to high specialization, provide support for chronic condition management, and ensure continuity of care through multidisciplinary collaboration, serving as a vital resource for urgent healthcare services [6,7,8]. In recent years, there have been many telemedicine initiatives, but too often, they are traced back to experiments, prototypes, and projects and ultimately characterized by high rates of failure [2]. The popularity of telemedicine peaked after the outbreak of COVID-19, and since then, the interest of healthcare systems in establishing effective telemedicine services has remained at high levels [9].

The field of vascular medicine encompasses a broad spectrum of acute and chronic circulatory diseases, affecting patients of any age but mainly the elderly or those with chronic comorbidities. Telemedicine is increasingly used in the field, but it is associated with several challenges, including the risk of misdiagnosis, issues concerning the security of personal healthcare information, and technological aspects in order to ensure the quality of the services. The aim of the present study was to investigate the views of experts and propose clinical practice recommendations on the potential role of telemedicine in vascular medicine. This work was based on a method used in a previous study published by the Italian Colon-Proctology Expert Group [2].

## 2. Materials and Methods

A literature search was performed using MEDLINE, PubMed, the Cochrane Database of Systematic Reviews, and the Cochrane Central Register of Controlled Trials from January 1990 until September 2022. The search strategy included the following keyword combinations: peripheral arterial disease/PAD AND wearable, venous diseases AND wearable, venous diseases AND telemedicine, capillaroscopy AND digital health, peripheral arterial disease AND artificial intelligence/machine learning, peripheral arterial disease AND big data, and telemedicine or telehealth or teleconsultation AND vascular disease.

After balancing the evidence of the literature review and clinical experience, group discussion led to shared opinions about recommendations for using telemedicine in the treatment of vascular diseases. In the absence of data from Oxford Level I to IV studies [10], the guided development group, composed of the steering committee and external advisors, produced a final list of clinical practice recommendations (CPRs). The group worked via emails and teleconferences and was responsible for suggesting the different topics to be incorporated and finalizing the items after thorough discussion. Thirty-eight international experts (based on previously published research and clinical experience in vascular medicine) were invited to join the e-consensus. The methodology was derived from the RAND/UCLA appropriateness method [11], an established approach previously used in the field [12,13]. 

Fifty-four CPRs were displayed electronically using an online platform (Google Form) under four subheadings: “Utility of telemedicine”, “Feasibility of telemedicine in vascular medicine”, “Sensitivity of telemedicine in vascular medicine”, and “Application of telemedicine in Vascular Medicine”. Moreover, 13 additional statements were presented as “Clinical Practice Recommendations”. In total, 67 CRPs were included.

For each statement, the panelists were queried with the following question: “Does the suggested recommendation result in anticipated health benefits (such as enhanced patient experience and functional capacity) that outweigh the anticipated negative consequences of its implementation (such as increased morbidity, anxiety, or denial of an investigation or treatment)”? A linear analog scale ranging from 1 to 10 was used for the responses in order to assess views on the benefit-to-harm ratio. On this scale, a score of 1 to 3 indicated that the panelist anticipated the harm of introducing the recommendation to greatly outweigh the expected benefits, whereas a score of 7 to 9 suggested that the expected benefits would significantly outweigh the anticipated harm. A middle rating, falling within the range of 4 to 6, could indicate either an assessment that the advantages and disadvantages were deemed equivalent or that the panelist was unable to render a definitive judgment regarding the recommendation.

The responses were analyzed as reported by the RAND/UCLA guidance, with each recommendation classified as “appropriate”, “uncertain”, or “inappropriate”, according to the panelists’ median score and the level of disagreement. Statements with median scores in the range of 1 to 3 were classified as inappropriate, those in the range of 4 to 6 as uncertain, and those in the range of 7 to 9 as appropriate. “Disagreement” implied the absence of consensus because of polarization, defined as more than 8 votes of the indication in each extreme for a sample of 32 panelists [11]. Regardless of the median score, all indications rated with “disagreement” were classified as “uncertain”. A second round of consensus was performed to mitigate variation using the same methodology. Only statements rated “uncertain” (i.e., having a panel median of 4–6, or any median with disagreement) were revisited and resubmitted for voting.

## 3. Results

Of the 38 invited experts, 32 joined the e-consensus. All participants involved in the first and second rounds were doctors working in academic or teaching hospitals.

### 3.1. Round 1

The results of Round 1 are summarized in Table 1 and presented below under each subheading.

#### 3.1.1. Utility of Telemedicine (Statements 1–11) 

Nine statements were rated as appropriate and two as uncertain. The highest median score was 10, for two statements assessing the usefulness of teleconsultation during the SARS-CoV-2 pandemic and in the case of geographical areas that are difficult to assess. Furthermore, five statements were rated with 8 or 9; these primarily concerned the usefulness of telemedicine in estimating the quality of life of vascular patients and decreasing waiting times, as well as the practical aspects of telemedicine, such as the need for payment and the possibility of written advice by chat/email. Two statements were rated as uncertain. More specifically, one of these referred to the possibility of scheduling surgery after a teleconsultation, and the other involved the need to administer the advice and/or prescriptions by verbal communication at the end of the teleconsultation.

#### 3.1.2. Feasibility of Telemedicine in Vascular Medicine (12–31) 

Good consensus (a median score of 9) was achieved for three statements, namely, “All third referral centers should have a teleconsultation system”, “It is recommended to perform a vascular medicine teleconsultation only with a vascular medicine expert” (reinforced by a statement rated 8, “A minimum level of 3–5 years of vascular medicine clinical experience is required to perform teleconsultations”), and “For the doctor it is recommended to use a sufficiently large screen (laptop or desktop PC) rather than a smartphone”. There were six statements that were rated as uncertain. The two lowest scoring, with a score of 4 (close to inappropriate), stated that “A vascular medicine teleconsultation with a general doctor is recommended” and “The post-surgical vascular medicine consultation can be performed via remote support”. Finally, one statement was considered inappropriate, which was that “Telemedicine and its potential can only be exploited in the period of the SARS-CoV-2 pandemic”. The panel’s answer was a clear “no”. 

#### 3.1.3. Sensitivity of Telemedicine in Vascular Medicine (32–38) 

The highest median value (10) was reached for the statement “Performing a teleconsultation with the help of a video support is recommended (i.e., the video call is superior to the voice call)”. The statement “Before a teleconsultation it is mandatory to get an appropriate informed consent from the patient” was rated with a median score of 9, and “The teleconsultation is useful to stratify patients according to the level of urgency with which they have to undergo a conventional outpatient consultation” had a median score of 8. Three statements were evaluated as uncertain, mainly based on the risk of losing “person-by-person” contact with patients and the risk of misdiagnosis.

#### 3.1.4. Application of Telemedicine in Vascular Medicine (39–54) 

Strong agreement was reached on the statement “It is always necessary to re-evaluate the patient with a conventional consultation after a teleconsultation before any surgical treatment”, good agreement (a median score of 8) about the use of teleconsultation in the evaluation of chronic venous insufficiency and Raynaud’s phenomenon, and less strong agreement (a median score of 7) about vascular ulcers. The panel was uncertain about the usefulness of teleconsultation in the diagnosis of Buerger’s disease, peripheral arterial disease (PAD), and critical limb ischemia (CLI) and rated as inappropriate the use of teleconsultation for the diagnosis and management of abdominal aortic aneurysm.

#### 3.1.5. Free-Text Statements (40–42)

Three statements from Section 3.1.4 (40–42) were formulated as free-text questions, as follows: “The teleconsultation in vascular medicine is applicable in the diagnosis and management of (INSERT SUGGESTION)”, without a pre-fixed answer. The majority of the panelists suggested PAD (10 answers), post-thrombotic syndrome (six answers), Raynaud’s phenomenon (five answers), chronic venous insufficiency (three answers), and chronic venous disease (three answers), but other answers included intermittent claudication, diabetic foot, venous ulcers, vascular acrosyndromes, varicose veins, lymphedema, critical leg ischemia, Buerger’s disease, and atypical limb pain. Some panelists commented that telemedicine is applicable for “follow-up only”, “follow up of non-complicated cases”, “triage/before in-person visits”, or as “first advice, if in-person visit is not possible”.

#### 3.1.6. Clinical Practice Recommendation (1–13)

The statements regarding the applicability of teleconsultation in vascular medicine as a screening tool, for checking the effectiveness of conservative medical therapy, for the evaluation of anticoagulant therapy in patients who had a deep vein thrombosis (DVT) or pulmonary embolism (PE), and for checking the effectiveness of medical therapy and certain essential technical aspects to ensure the robustness of the platform were deemed appropriate, with a median score of 8. Moreover, its applicability for evaluating superficial vein thrombosis in patients who have undergone a recent Color-coded 2-dimensional Doppler was rated as appropriate. However, the panel was uncertain about its applicability for the diagnosis and monitoring of PAD after Color-coded 2-dimensional Doppler and about the use of social media and “nonspecific” platforms for teleconsultation.

### 3.2. Round 2

After reviewing the results gathered in the initial round, 11 additional statements were re-evaluated during the second round to minimize variability (Table 2). There was a strong agreement about the liability risks that teleconsultation may increase and the need for a dedicated insurance policy. Contrary to Round 1, in Round 2, the statement “The teleconsultation in vascular medicine is applicable in the diagnosis of PAD” was deemed appropriate (a median score of 7) as was “The teleconsultation in vascular medicine enhances the follow-up of PAD” (median score 9). The statements regarding the cost of teleconsultation in relation to conventional consultation were rated as uncertain.

## 4. Discussion

During the COVID-19 pandemic, telemedicine was demonstrated to be significantly valuable for vascular medicine doctors, allowing continuity of care with several benefits, such as accessibility and rapidity. It should be noted that telemedicine does not replace the conventional health service in terms of the physician–patient relationship but integrates it to enhance and improve effectiveness, efficiency, and appropriateness [14]. It is a novel approach to medicine in which the information to be transmitted may involve the voice, images, numbers, written data, or instruments moved at a distance. It must also comply with all the rights and obligations that regulate any medical act. It is a change in how we perceive healthcare; it guarantees greater speed but not at the cost of lower quality [15]. The panelists agreed that the scope of telemedicine extends beyond the COVID-19 emergency. However, given that the transition to a telehealth model in recent years has been rapid, there are several undefined challenges (e.g., reimbursement, the doctor–patient relationship, and appropriate technology platforms) that should be gradually clarified.

An important finding of our study is the views of the panelists on issues regarding telehealth services’ professional liability, reimbursement and insurance requisites, and medicolegal implications. More specifically, teleconsultations are recommended to be compensated on a regular basis, and advice and prescriptions should be included in a formal report sent to the patient, along with a receipt. The majority of the panelists opposed the dissemination of advice and prescriptions solely through verbal communication or direct chat, even though the latter option may still have its legal value. There was uncertainty regarding the cost of the teleconsultations (median scores of 5 and 6 in two relevant statements). Furthermore, the panelists expressed the view that obtaining proper remote informed consent is essential to record the entire visit and safeguard data confidentiality. Nevertheless, two primary challenges arise: (a) identifying suitable archiving protocols for recordings and (b) determining the permissibility of using these recordings for legal medicine issues. Several of those issues are addressed in the ATA Policy Principles, which highlight the need for broad coverage of all forms of telehealth services [16]; however, the specific policies depend on the health insurance system of each country [17]. According to the ATA, apart from physicians, all healthcare providers at all levels must be able to engage across telehealth care teams [16]. In addition, it is imperative to prioritize patient privacy and data security to ensure the viability of telehealth. Hence, regulation should mitigate cybersecurity risks and provide patient confidentiality [16].

Most participants considered uncertain the possibility of performing the first visit remotely (a median score of 5). However, teleconsultation for screening or pre-hospitalization purposes, such as evaluating the need for diagnostic testing before an in-person appointment or follow-up (e.g., to check the effectiveness of conservative therapy), was deemed appropriate. Indeed, the utilization of teleconsultation to evaluate and stratify the urgency of care, determining whether a conventional outpatient consultation is necessary, was recognized as appropriate. The voting results revealed a strong recommendation for patient reassessment shortly after a teleconsultation, particularly for surgical candidates. These findings underscore the crucial role of physical examination in vascular medicine, emphasizing the concern among specialists about the potential for misdiagnosis (a median score of 8). An incorrect or delayed diagnosis after an unperformed physical examination, depending on the nature of the condition, may result in a range of minor to severe consequences. For example, a delayed cancer diagnosis can have a profound impact on a patient’s life.

Regarding the nature of the diseases that could be assessed, teleconsultation was not recommended for the diagnosis and management of aortic aneurysm; it was instead deemed appropriate for the diagnosis and management of evaluation of chronic venous insufficiency and Raynaud’s phenomenon, and of vascular ulcers, though there was less strong agreement about this (a median score of 7). The above diseases may have been recommended as appropriate for telemedicine evaluation because of the ease of diagnosis facilitated by high-definition pictures, as well as the typical localization of the disease. In the first round, the results revealed uncertainty about the use of telemedicine for diagnosis and monitoring of PAD after Color-coded 2-dimensional Doppler. However, in the second round, the use of telemedicine for diagnosis, but mostly for follow-up, of PAD was rated as appropriate.

In the first round, there was uncertainty about using social media and “nonspecific” platforms for teleconsultation. Those doubts may be reduced after increased experience in the use of telemedicine. In the second round, after some clarifications, the statement concerning the use of telemedicine on any conference call platform was rated as appropriate (a median score of 9), the only limits being solid encryption and data privacy protection. Nevertheless, using social media messaging for patient communications was deemed uncertain. 

Teleconsultation was suggested as a means to connect with specialized centers equipped with expert teams dedicated to managing vascular diseases. This approach would facilitate virtual hub-and-spoke discussions of challenging cases involving the transmission of pertinent documentation to tertiary centers to reach outcome decisions. Ideally, a teleconsultation system should be accessible in all tertiary centers, and future studies should aim to assess the effectiveness of a telemedicine program. To this end, a comprehensive standardized checklist should be developed with all the main requirements of such systems [18]. Besides teleconsultation, the teleproctoring of challenging vascular surgery cases with the aid of experienced surgeon proctors from specialized institutions is another appealing application of digital health technologies [19]. Such procedures were successfully reported in the field of vascular surgery during the COVID-19 pandemic [19]. A broader use of teleproctoring could be of benefit for remote areas and non-experienced centers [7]. Notably, the appropriate technical support is essential [19].

The present study has some limitations. The primary goal was to shape the framework for understanding and preventing harm caused by the reckless use of telemedicine in vascular medicine. Further evidence is required to define its role in this context, and clinical studies are needed to clarify what works and what does not. Another limitation could be that, despite being chosen based on their publication track record in the field of vascular medicine, the participants had limited overall experience with telemedicine at the time of the study. Therefore, certain results may have reflected a more skeptical view concerning the suitability of telemedicine in a specialty where objective examination is necessary. Finally, the achievement of a higher level of agreement among the panelists might have been hampered due to several concerns that fueled skepticism. These concerns included the potential existence of a digital gap among patients (i.e., insufficient access to required equipment or technological expertise) as well as technical challenges in using the system or its lack of user-friendliness. Initiatives to extend high-speed broadband internet access to underserved communities and to implement outreach programs are required to prevent telehealth from worsening health disparities [20]. Other potential concerns of the panelists included obstacles related to reimbursement and licensing, apprehensions about litigation, and ethical issues of transparency, privacy, and confidentiality [21,22,23]. For example, it has been shown that many health applications exhibit inconsistent privacy practices, with a considerable portion lacking explicit privacy policies [24]. Measures aiming to regulate privacy policies and enable patients to make informed decisions are greatly anticipated.

## 5. Conclusions

In conclusion, our study underlines the points of agreement and those that raise concerns regarding the use of telemedicine in vascular medicine. It may provide a framework in order to incorporate the consistent and careful use of telemedicine in the field of vascular medicine. Several issues need to be resolved, namely, the standardization of infrastructure, logistics, and legislation to guarantee a smooth healthcare service while preserving the integral patient–physician relationship. Given that technological advances are continuous and will doubtless be incorporated into medicine, it is of paramount importance to recognize the advantages and the limits of healthcare technologies.

## Figures and Tables

**Table 1:** Results of voting for Clinical Practice Recommendations in Round 1.

Telemedicine Applications in Vascular Medicine—Round 1						
Statement Number	Clinical Practice Recommendations	Median Score	Score Distribution (N)			Decision
			≤3	4–6	≥7	
**Utility of Telemedicine**						
1	Telemedicine may facilitate the management of vascular patients during the SARS-COV-2 pandemic, allowing continuity of care	10	0	3	29	Appropriate
7	Telemedicine reduces the distance to areas that are difficult to access or geographically distant	10	0	0	32	Appropriate
3	Teleconsultation has the value of a specialist consultation, and as such should be regularly paid	9	1	6	25	Appropriate
5	Telemedicine is a useful tool to estimate the quality of life of vascular patients for scientific purposes (e.g., PROMS—Patient Reported Outcome Measures)	9	0	2	30	Appropriate
2	Telemedicine, associated with conventional outpatient activities, can guarantee a reduction in waiting times	8	0	4	28	Appropriate
4	A pre-interview via teleconsultation (e.g., specifying preoperative investigations) is useful before the conventional consultation	8	2	7	23	Appropriate
10	At the end of the teleconsultation, the advice and/or prescriptions can be directly written in chat or sent by e-mail	8	1	6	25	Appropriate
6	Telemedicine is a useful tool for performing pre hospitalization (preoperative assessment exams)	7	1	12	19	Appropriate
8	In case of impossibility or interruption of the connection due to technical or unexpected problems with facilitator, the visit can be carried out or completed by telephone	7	2	11	19	Appropriate
11	It is possible to schedule surgery after a teleconsultation	5	11	14	7	Uncertain
9	At the end of the teleconsultation, the advice and/or prescriptions can be only administered by a verbal communication	4	16	9	7	Uncertain
**Feasibility of Telemedicine in Vascular Medicine**						
19	It is recommended to perform a Vascular Medicine teleconsultation only with a Vascular Medicine expert	9	1	6	25	Appropriate
25	For the doctor it is recommended to use a sufficiently large screen (laptop or desktop PC) rather than a smartphone	9	1	10	19	Appropriate
30	All third referral centers should have a teleconsultation system	9	4	5	22	Appropriate
12	The teleconsultation (consultation between doctor and patient) is applicable in the Vascular Medicine field	8	0	5	27	Appropriate
13	The tele-expertise consultation between the Vascular Medicine expert and the doctor who visited the patient) is applicable in the Vascular Medicine field	8	2	3	27	Appropriate
14	Telemonitoring (detection and sending by the patient of preestablished parameters considered crucials for a rapid re-evaluation) is applicable in the Vascular Medicine field	8	0	8	24	Appropriate
16	A minimum level of 3–5 years of Vascular Medicine clinical experience is required to perform teleconsultations	8	0	7	25	Appropriate
17	Training on how to use platforms and informatic systems to support telemedicine is essential, regardless of the clinical experience of the Vascular Medicine expert	8	0	8	24	Appropriate
18	Training on how to perform and conduct a visit or consultation in telemedicine is essential, regardless of the clinical experience of the Vascular Medicine expert	8	0	8	24	Appropriate
24	To perform teleconsultation with less technological patients, it is appropriate to identify a “key contact” that would act as a technological facilitator for the patient or family	8	1	6	23	Appropriate
29	Photos/videos sent by the patient during the teleconsultation can be helpful for the doctor	8	2	4	24	Appropriate
22	The Vascular Medicine consultation (a control excluding post-operative follow-up) can be performed via remote support	7	3	11	16	Appropriate
27	With the consent of the patient, it is recommended to record the teleconsultation	7	5	10	16	Appropriate
26	The use of a screen that allows Full-HD or 4K viewing is recommended for the doctor	6	2	13	15	Uncertain
28	At the end of the teleconsultation, a screenshot containing the patient image and the written report may be sufficient as a guarantee of performance	6	6	13	12	Uncertain
21	The first Vascular Medicine consultation can be performed via remote support	5	7	16	8	Uncertain
31	The services provided in telemedicine share the same specific characteristics of the professional liability and insurance of a conventional consultation	5	6	10	14	Uncertain
20	A Vascular Medicine teleconsultation with a general doctor is recommended	4	10	19	3	Uncertain
23	The post-surgical Vascular Medicine consultation can be performed via remote support	4	12	13	5	Uncertain
15	Telemedicine and its potential can only be exploited in the period of the Sars-Cov-2 pandemic	2	23	7	2	Inappropriate
**Sensitivity of Telemedicine in Vascular Medicine**						
38	Performing a teleconsultation with the help of a video support is recommended (i.e., the video call is superior to the voice call)	10	5	3	24	Appropriate
37	Before a teleconsultation it is mandatory to get an appropriate informed consent from the patient	9	6	5	21	Appropriate
34	The teleconsultation is useful to stratify patients according to the level of urgency with which they have to undergo a conventional outpatient consultation	8	4	2	25	Appropriate
36	The medical history collected during a teleconsultation is completely comparable to that collected during a conventional consultation	7	8	7	17	Appropriate
33	The teleconsultation could increase the number of misdiagnosis for cancers	6	8	10	13	Uncertain
35	The number of patients lost to follow-up after a teleconsultation can be worrying	6	10	12	9	Uncertain
32	The teleconsultation could increase the number of misdiagnosis	5	7	13	10	Uncertain
**Application of Telemedicine in Vascular Medicine**						
51	It is always necessary to re-evaluate the patient with a conventional consultation after a teleconsultation before any surgical treatment	9	6	2	24	Appropriate
46	The teleconsultation in vascular medicine is applicable in the diagnosis and management Chronic Venous Insufficiency	8	6	2	23	Appropriate
48	The teleconsultation with specialized centers is useful in the management of patients affected by Raynaud Phenomenon	8	4	7	20	Appropriate
49	The teleconsultation is applicable in the management of patients with Vascular Ulcers	7	6	8	17	Appropriate
54	In telemedicine it is necessary to use dedicated video-call platforms that are not included into the ‘social’ category	7	5	10	17	Appropriate
39	Teleconsultation should have the same cost as a conventional specialist consultation	6	7	10	15	Uncertain
47	The teleconsultation with specialized centers is useful in the management of patients affected by Buerger Disease	6	5	11	15	Uncertain
52	The time interval for a control after a teleconsultation should be shorter than that after a conventional consultation	6	8	11	13	Uncertain
44	The teleconsultation in vascular medicine is applicable in the diagnosis and management PAD	5	12	7	12	Uncertain
45	The teleconsultation in vascular medicine is applicable in the diagnosis and management of CLI	5	10	8	13	Uncertain
50	It is always necessary to re-evaluate the patient in a conventional consultation after a teleconsultation	5	9	14	8	Uncertain
53	In telemedicine it is allowed to use social tools for video-calls (e.g., Apple FaceTime, Facebook Messenger or WhatsApp, Zoom, Google Hangouts video, Skype)	5	14	7	11	Uncertain
43	The teleconsultation in vascular medicine is applicable in the diagnosis and management of Abdominal Aortic Aneurism	2	21	9	2	Inappropriate
**Clinical Practice Recommendations**						
1	The teleconsultation (between doctor and patient) is applicable in vascular medicine as a screening (e.g., to indicate diagnostic tests) prior to an outpatient consultation	8	5	2	25	Appropriate
3	Teleconsultation is routinely applicable for checking the effectiveness of conservative medical therapy	8	4	4	24	Appropriate
6	The teleconsultation is applicable for the evaluation of anti-coagulant therapy in patients who had a DVT or EP	8	5	7	20	Appropriate
9	The teleconsultation is applicable for checking the effectiveness of medical therapy	8	3	3	26	Appropriate
10	The teleconsultation should be carried out on appropriate platforms recognized by the national health system	8	2	5	25	Appropriate
12	At the end of the teleconsultation, the advice and/or prescriptions should be necessarily written directly in chat or sent by e-mail	8	2	6	24	Appropriate
13	A formal receipt should be released by the supplying system as a guarantee of regularity after the teleconsultation	8	2	7	13	Appropriate
7	The teleconsultation is applicable for the evaluation of superficial vein thrombosis in patients who have undergone a recent Color-coded 2-dimensional Doppler	7	6	10	16	Appropriate
2	The teleconsultation (between specialist and doctor who visited the patient) is indicated only if the interlocutor is a vascular medicine specialist	6	9	10	13	Uncertain
8	The teleconsultation is applicable for the diagnosis and monitoring of PAD after Color-coded 2-dimensional Doppler	6	8	9	15	Uncertain
4	The teleconsultation is applicable for routine post-operative controls	5	8	15	9	Uncertain
5	The cost of a teleconsultation should be lower than that of a conventional consultation	5	13	8	11	Uncertain
11	Social media (e.g., Apple FaceTime, Facebook Messenger or WhatsApp, Zoom, Google Hangouts video, Skype) can be used for patient communications regarding their status	5	11	12	9	Uncertain

**Table 2:** Results of voting for Clinical Practice Recommendations in Round 2.

Telemedicine Applications in Vascular Medicine—Round 2						
Statement Number	Clinical Practice Recommendations	Median Score	Score Distribution (N)			Decision
			≤3	4–6	≥7	
1	In telemedicine it is allowed to use any conference call platform, the only limits are a strong encryption and protection of privacy of data	9	1	2	15	Appropriate
2	The cost of a teleconsultation should be lower than that of a conventional consultation	5	7	4	7	Uncertain
3	The cost of a teleconsultation should be the same of a conventional consultation	6	4	5	9	Uncertain
4	The cost of a teleconsultation should be higher than that of a conventional consultation	1	16	1	1	Inappropriate
5	The services provided in telemedicine increase the risks of professional liability	8	1	4	13	Appropriate
6	The services provided in telemedicine need dedicated insurance policy	9	0	3	15	Appropriate
7	The teleconsultation is a useful tool for the initial screening of vascular diseases and defining priority of the in-person visit	9	0	1	17	Appropriate
8	The teleconsultation in vascular medicine is applicable in the diagnosis of PAD	7	4	5	9	Appropriate
9	The teleconsultation in vascular medicine enhances the follow up of PAD	9	1	0	17	Appropriate
10	Social media messaging (e.g., Facebook Messenger or WhatsApp, Telegram) can be used for patient communications regarding their status	4	9	3	6	Uncertain
11	At the end of the teleconsultation the written report is always needed	10	0	2	16	Appropriate

## Data Availability

Data are available upon request.
